# Linker region is required for efficient nuclear localization of polynucleotide kinase phosphatase

**DOI:** 10.1371/journal.pone.0239404

**Published:** 2020-09-24

**Authors:** Kaima Tsukada, Yoshihisa Matsumoto, Mikio Shimada

**Affiliations:** Laboratory for Advanced Nuclear Energy, Institute of Innovative Research, Tokyo Institute of Technology, Tokyo, Japan; University of South Alabama Mitchell Cancer Institute, UNITED STATES

## Abstract

Polynucleotide kinase phosphatase (PNKP) is a DNA repair factor with dual enzymatic functions, *i*.*e*., phosphorylation of 5’-end and dephosphorylation of 3’-end, which are prerequisites for DNA ligation and, thus, is involved in multiple DNA repair pathways, *i*.*e*., base excision repair, single-strand break repair and double-strand break repair through non-homologous end joining. Mutations in PNKP gene causes inherited diseases, such as microcephaly and seizure (MCSZ) by neural developmental failure and ataxia with oculomotor apraxia 4 (AOA4) and Charcot-Marie-Tooth disease 2B2 (CMT2B2) by neurodegeneration. PNKP consists of the Forkhead-associated (FHA) domain, linker region, phosphatase domain and kinase domain. Although the functional importance of PNKP interaction with XRCC1 and XRCC4 through the FHA domain and that of phosphatase and kinase enzyme activities have been well established, little is known about the function of linker region. In this study, we identified a functional putative nuclear localization signal (NLS) of PNKP located in the linker region, and showed that lysine 138 (K138), arginine 139 (R139) and arginine 141 (R141) residues therein are critically important for nuclear localization. Furthermore, double mutant of K138A and R35A, the latter of which mutates arginine 35, central amino acid of FHA domain, showed additive effect on nuclear localization, indicating that the FHA domain as well as the NLS is important for PNKP nuclear localization. Thus, this study revealed two distinct mechanisms regulating nuclear localization and subnuclear distribution of PNKP. These findings would contribute to deeper understanding of a variety of DNA repair pathway, *i*.*e*., base excision repair, single-strand break repair and double-strand break repair.

## Introduction

Genotoxic stresses, such as exposure to the ionizing radiation (IR) and chemical compound, induce a variety of damages on genomic DNA, such as base damage, DNA single-strand breaks (SSBs) and DNA double-strand breaks (DSBs). XRCC1 is a key scaffold protein in base excision repair (BER) and SSB repair (SSBR) [1–4]. Because of base damage is frequent DNA damage arising from oxidative stress in cellular level, suppression of XRCC1 results in accumulation of DNA damage and genomic instability [5]. Meanwhile, XRCC4 is a key scaffold protein, involved in non-homologous end joining (NHEJ) for DSB repair (DSBR) [6,7]. In the NHEJ repair pathway, following phosphorylation of XRCC4 by CK2, other NHEJ factors, XLF, PAXX, and Ligase IV are accumulated to the DNA ends to form complex with XRCC4 [7].

Polynucleotide kinase phosphatase (PNKP) has dual enzymatic functions, *i*.*e*., phosphatase activity to remove phosphate groups from 3’-ends of DNA, if present, and kinase activity to add phosphate groups to 5’-ends [8,9]. Since the presence of phosphate group at 5’-end and its absence at 3’-end are prerequisites for DNA ligation, PNKP is involved in multiple DNA repair pathways, *i*.*e*., BER, SSBR and DSBR through NHEJ. In fact, mutations in PNKP gene cause inherited diseases accompanying severe neural developmental failure, such as microcephaly and seizure (MCSZ), and neurodegeneration such as ataxia with oculomotor apraxia 4 (AOA4) and Charcot-Marie-Tooth disease 2B2 (CMT2B2) [10–14]. Furthermore, in mouse model, defect of PNKP, as well as XRCC1, in central nervous system result in massive DNA damage and apoptosis in neural progenitors leading to the neural developmental failure such as neurodegenerative disease and microcephaly [15–17].

PNKP consists of the Forkhead-associated (FHA) domain, linker region, phosphatase domain, and kinase domain, which are aligned from N-terminus to C-terminus [18].

The phosphatase and kinase domains are shown to be important for regulation of DNA ends processing [2,19]. FHA domain is shown to be a binding module for peptide containing phosphorylated amino acids, especially phosphorylated threonine [20]. Indeed, PNKP associates with XRCC1 and XRCC4 in a manner dependent on threonine phosphorylation (threonine 519 and threonine 233, respectively) via FHA domain [6,21]. In contrast to the accumulated knowledge on the function of FHA and catalytic domains, little is known about the function of the linker region. Serine 114 and 126 in the linker region are phosphorylated by ataxia telangiectasia mutated (ATM) and DNA-dependent protein kinase catalytic subunit (DNA-PKcs) [22,23]. It was also shown that the phosphorylation of PNKP at serine 114 and 126 by ATM prevents proteasomal degradation of PNKP [24]. Nevertheless, the function of the remaining of the linker region is mostly unknown, in spite of the high degree of conservation across mammalian species.

Nuclear transportation depends on association with importin α and nuclear localization signal (NLS). NLS is conventionally classified into monopartite NLS consisting of a single cluster of basic residues and bipartite NLS consisting of two clusters of basic residues [25,26]. In addition, some proteins, such as the epidermal growth factor receptor (EGFR) and pericentrin contain tripartite NLS consisting of three clusters of basic residues [27,28]. All types of NLS sequences are recognized by importin α and translocate with importin β through nuclear pore complex into the nucleus.

In this study, we identified functional putative NLS, which is located between amino acid 137–142 in the linker region of PNKP, and the key residues lysine 138, arginine 139 and arginine 141 therein, which are essential for nuclear localization of PNKP. Additionally, our data suggest possibility that FHA domain might regulate distribution to nuclear components such as nucleolus.

## Materials and methods

### Cell culture

The human embryonic kidney cell line HEK293, and the human osteosarcoma cell line U2OS were obtained from American Type Culture Collection and maintained in Dulbecco’s modified Eagle’s medium (DMEM; Nacalai Tesque, Inc.) supplemented with 10% v/v fetal bovine serum (FBS; Hyclone, GE Healthcare) and penicillin/streptomycin (Nacalai Tesque, Inc.) at 37°C in humidified atmosphere containing 5% CO_2_. All cell lines were tested for mycoplasma contamination using e-Myco^TM^ mycoplasma detection PCR kit (iNtRON Biotechnology, Inc., cat# 25235).

### Construction of plasmid DNA, mutagenesis, and cDNA transfection

pEGFP-C1 and mCherry2-C1 was obtained from Clontech and Addgene, respectively. Full-length human PNKP and Fibrillarin cDNAs were obtained by PCR from the cDNA pool of U2OS cells and inserted into pEGFP-C1 and mCherry2-C1, respectively. Deletions and point mutations in PNKP (D1 (deletion of amino acids 2–110), D2 (deletion of amino acids 111–145), D3 (deletion of amino acids 146–337), D4 (deletion of amino acids 338–521), Δ137–142 (deletion of amino acids 137–142), R35A, K137A, K138A, R139A, R141A, K142A, R35A/K138A, and K138A/R139A) were introduced using PrimeSTAR mutagenesis basal kit (Takara Bio, cat# R046A), according to the manufacturers’ instruction. All primers for point and site-directed mutagenesis were designed using the Agilent QuikChange primer design program (S1 Table). All DNA constructs were verified by DNA sequence analysis. For cDNA transfections, PEI-MAX (Polysciences, Inc., cat# 24765) or Lipofectamine 3000 (Invitrogen) were used according to the manufacturers’ instruction.

### Protein depletion by siRNA transfection

For siRNA transfection, Lipofectamine RNAiMAX (Invitrogen) was used according to the manufacturer’s instruction. The siRNAs were used at a final concentration of 50 nM at 48h-72h prior to the experiment. The siRNA sequence of siLUC (targeting luciferase as control: Dharmacon, cat# D-001210-02-20) and siPNKP 3’-UTR (targeting 3’-untranslated region of PNKP gene) are 5’- TAAGGCTATGAAGAGATAC -3’ and 5’- CACAATAAACGCTGTTTCTCC -3’, respectively.

### Live-cell imaging

U2OS cells were grown on glass-bottom 35mm dish (Matsunami Glass Ind., Ltd.) and GFP- or mCherry-tagged cDNA were transfected into the cells at 2 days before observation using the method as described above. On the subsequent day, culture media containing the transfection reagents were replaced with normal culture media without transfection reagents. At the day of observation, normal culture media were replaced with phenol red-free DMEM (Nacalai Tesque, Inc.) supplemented with 10% FBS and Hoechst 33342 (Dojindo Molecular Technologies, Inc.) were added for staining DNA 30 min prior to observation. Zeiss LSM880 (Carl Zeiss) were used for live-cell observation and capturing pictures. For the measurement of green fluorescence intensity (GFI) in the nucleus and the cytoplasm, we randomly selected a certain size of areas in the nucleus and the cytoplasm of each cell, and calculated the average values of GFI using Image J software (NIH). The nucleus/cytoplasm ratio (N/C ratio) was calculated by Microsoft Excel (Microsoft Co., Ltd.). In all live-cell imaging experiments, U2OS cells were used and at least 150 cells were scored.

### SDS-PAGE and western blotting

Cells were lysed in a radioimmunoprecipitation assay (RIPA) buffer (50 mM Tris HCl, pH 8.0; 250 mM NaCl; 25 mM ethylenediaminetetraacetic acid (EDTA); 0.5% Triton X-100; 0.5% sodium dodecyl sulfate (SDS); and 0.5% sodium deoxycholate) containing protease inhibitor cocktail (Nacalai Tesque, Inc., cat# 25955–11) and phosphatase inhibitor cocktail (Nacalai Tesque, Inc., cat# 07575–51), and the protein concentration was measured by a bicinchoninic acid (BCA) assay kit (Takara Bio) using bovine serum albumin as the standard. In all experiment, 20 μg of protein was loaded onto SDS polyacrylamide gel electrophoresis (SDS-PAGE) plates. The proteins were electrophoresed at 30 mA/gel plate for 1–1.5 h, and transferred onto a polyvinylidene fluoride (PVDF) membrane at 100 V for 1.5 h. Next, the PVDF membrane was blocked with either 2% BSA/TBS-T (tris-buffered saline and Tween 20) or 5% skim milk/TBS-T for 1 h at room temperature on a shaker. For primary antibody reactions, the following primary antibodies were used for 1–4 h at room temperature: PNKP (rabbit, 1:1000, Novus, cat# NBP1-87257), XRCC1 (mouse, 1:1000, Invitrogen, cat# MA5-13412), XRCC4 (rabbit, 1:1000, generated in our laboratory [29]), GAPDH (mouse, 1:10000, EMD Millipore Corp., cat# MAB374), GFP (mouse, 1:5000, Nacalai Tesque, cat# 04363–24), KAP-1 (rabbit, 1:1000, abcam, cat# ab10483). The PVDF membrane was washed three times with TBS-T. For secondary antibody reactions, horseradish peroxidase (HRP)-conjugated rabbit or mouse antibodies (Dako, cat# P0399 or P0447, respectively) were used for 1 h at room temperature. After five times washing with TBS-T, the membrane was developed by enhanced chemiluminescence (LI-COR, Biosciences) and detected by C-digit (LI-COR, Biosciences).

### Immunofluorescence

Cells were grown on glass coverslips and incubated for 30 min or 6 h after 2 Gy γ-ray exposure. After induction of DNA damages, cells were fixed with 4% formaldehyde for 15 min at 4°C. Cells were subsequently permeabilized with PBS containing 0.2% Triton X-100 for 5 min at 4°C. Following 30 min of blocking in PBS supplemented with 2% BSA, primary antibody reactions were performed in PBS-T supplemented with 1% BSA for 2 h at room temperature. Cells were washed three times with PBS, and secondary antibody reactions were performed in PBS-T supplemented with 1% BSA for 1 h at room temperature in the dark. After five times washing with PBS, coverslips were mounted in mounting medium (Dako) containing the nuclear staining dye 4’,6-diamidino-2-phenylindole dihydrochloride (DAPI), and allowed to dry for 2 h at room temperature in the dark. For a primary antibody, γ-H2AX mouse antibody (Merck Millipore, JBW301) was used at 1:1000 dilution. Alexa Fluor 594-conjugated mouse secondary antibody (Invitrogen, cat# A32742) was used at 1:2000 dilution. For the measurement of SSB repair efficiency, cells were pre-treated with 10μM of Poly (ADP-ribose) glycohydrolase inhibitor (PARGi, TOCRIS Bio-Techne, PDD 00017273) for 30 mins prior to IR exposure. As the primary reaction to detect SSBs, a PAN-ADP-ribose binding reagent (rabbit, Merck, cat# 9QQ12P) was used at 1:1000 dilution in PBS-T supplemented with 1% BSA. Alexa Fluor 594-conjugated rabbit secondary antibody (Invitrogen, cat# A32740) was used at 1:2000 dilution. The mean intensity of ADP-ribose in the nucleus stained with DAPI was measured by ImageJ software. At least 100 cells were counted, and calculated the average value of ADP-ribose intensity by Graphpad Prism 8 (GraphPad Software Inc.).

### Immunoprecipitations (IP)

For sample preparation for immunoprecipitations (IP), HEK293 cells were grown on 10 cm dish and washed twice in phosphate-buffered saline (PBS: Nacalai Tesque, Inc.), and lysed in lysis buffer (100 mM NaCl, 0.2% NP-40, 1 mM MgCl_2_, 10% glycerol, 50 mM Tris-HCl, pH 7.5), supplemented with protease and phosphatase inhibitor cocktail. After 30 min-incubation on the rotator at 4°C, lysates were cleared by the centrifugation 20,000 x g for 20 mins at 4°C. Next, lysates were incubated with 10 μl of GFP-Trap magnetic agarose beads (ChromoTek, GmbH) for 2 h with mixing on a rotator at 4°C. Lysates were then washed five times with lysis buffer, and eluted in 2X SDS sample buffer (125 mM Tris-HCl, pH 6.8, 4% SDS, 20% glycerol, 0.01% bromophenol blue, 5% 2-mercaptoethanol).

### Statistical analysis

Statistical analysis was performed using either GraphPad Prism 8 (GraphPad Software Inc.) or Microsoft Excel. Unpaired t-test or one-way ANOVA followed by Tukey post-hoc test was applied to analyze the statistical significance of difference between two or multiple experimental groups, respectively. Sample scales are indicated in figure legends. All experiments were performed at least twice. In all experiments: not significant (ns) is defined as p > 0.05, *: 0.01 < p ≦ 0.05; **: 0.005 < p ≦ 0.01; ***: 0.001 < p ≦ 0.005; ****: 0.0005 < p ≦ 0.001.

## Results and discussion

### Linker region of PNKP is required for efficient nuclear localization

**Fig 1:**
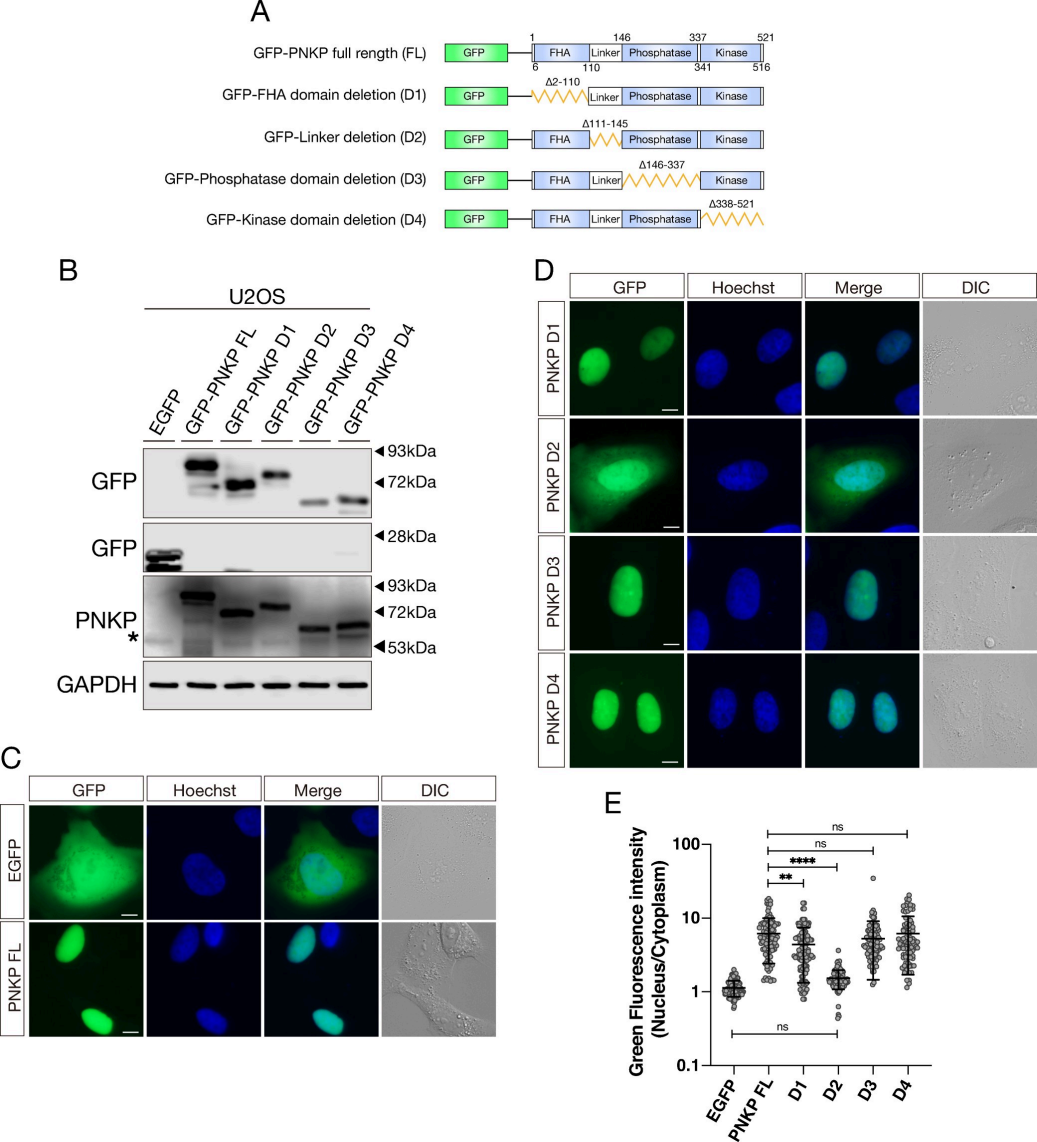
Linker region of PNKP is required for nuclear localization. (A) Schematic representation of the PNKP protein structure indicating the Forkhead-associated (FHA) domain, linker region, phosphatase domain, and kinase domain. Below is the schematic diagram of PNKP truncated mutants (D1-D4). (B) Confirmation of protein expression of GFP-PNKP constructs and endogenous PNKP in U2OS cells by western blot analysis. GFP-PNKP and endogenous PNKP was detected by anti-GFP and PNKP antibodies, respectively. Asterisk represents endogenous PNKP expression. GAPDH was used as loading control. (C) Representative live-cell images of GFP and GFP-tagged full-length (FL) PNKP, expressing in U2OS cells, with differential interference contrast (DIC) images. Scale bar indicates as 10μm. (D) Representative live-cell images of GFP-tagged truncated PNKP (D1-D4) mutants, expressing in U2OS cells, with DIC images. Scale bar indicates as 10μm. (E) Dot plot of the N/C ratio of GFI in indicated cDNA. At least 150 cells were analyzed and plotted. ns: not significant (p > 0.05); **: 0.005 < p ≦ 0.01; ****: 0.0005 < p ≦ 0.001.

PNKP is a dual functional protein and involved in variety of DNA repair pathways such as SSB and DSB repair. However, it is still unclear how is PNKP regulated and recruited to DNA repair sites. Therefore, we first attempted to investigate the regulation mechanism of PNKP. PNKP consists of FHA, linker, phosphatase and kinase domains (Fig 1A). To investigate how PNKP is recruited to the DNA damage sites, we first constructed cDNA expression vectors for green fluorescence protein (GFP)-tagged full-length (FL) PNKP cDNA and partially deleted mutants, indicated as FHA deletion (D1), linker deletion (D2), phosphatase deletion (D3) and kinase deletion (D4). Next, these cDNA expression vectors were transfected into human osteosarcoma cell line U2OS. Protein expression level of these constructs was confirmed by western blot analysis (Fig 1B). According to the live-cell imaging results, expression of FL, D1, D3, and D4 PNKP was dominantly observed in nucleus (Fig 1C and 1D). In contrast with FL PNKP, expression of D2 PNKP was clearly observed in the outside as well as the inside of nucleus (Fig 1D). We measured green fluorescence intensity (GFI) in the nucleus and the cytoplasm and calculated the nucleus/cytoplasm ratio (N/C ratio) and statistical significance of difference between FL PNKP and these deletion mutants (Fig 1E). D2 PNKP (Mean of column: 1.53) showed significantly smaller N/C ratio than FL PNKP (Mean of column: 6.119), although D1 PNKP (Mean of column: 4.293) also showed modestly but significantly reduced N/C ratio, suggesting mildly abrogated nuclear localization ability. On the other hand, D3 (Mean of column: 5.274) and D4 (Mean of column: 6.104) showed similar N/C ratio of GFI to FL PNKP. From these results, we concluded that the linker region is important for efficient nuclear localization of PNKP. Additionally, we inferred that the FHA domain might also contribute to the distribution of PNKP in nucleus, albeit to a lesser extent than the linker region.

### Lysine 138, arginine 139 and arginine 141 of PNKP are indispensable to localize into nucleus

**Fig 2:**
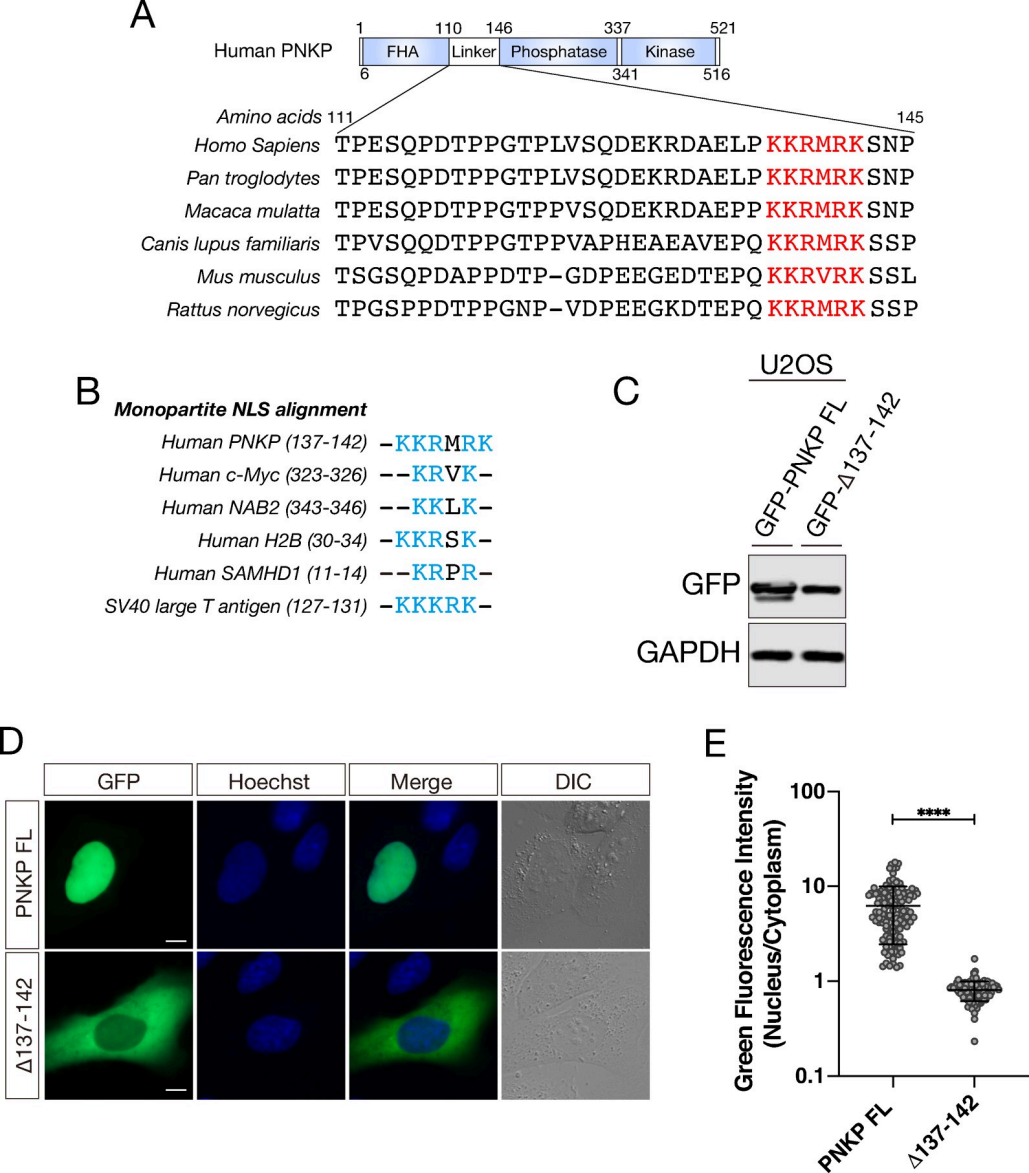
Amino acids 137–142 are essential for nuclear localization of PNKP. (A) Alignment of linker region PNKP amino acid sequence among a variety of mammalian species. Highly conserved amino acid sequence 137–142 were indicated by red. (B) Alignment of putative PNKP NLS and monopartite NLS motifs in other proteins. Positively charged amino acids were indicated by light blue. (C) Confirmation of protein expression of GFP-PNKP constructs in U2OS cells by western blot analysis. (D) Representative live-cell images of GFP-tagged FL PNKP and NLS (137–142 deletion)-deleted PNKP, expressing in U2OS cells, with DIC images. Scale bar indicates as 10μm. (E) Dot plot of the N/C ratio of GFI in FL and NLS deleted PNKP. At least 150 cells were analyzed and plotted. ****: 0.0005 < p ≦ 0.001.

Short specific amino acid motifs have an important role to localize proteins to cellular compartments including nucleus. NLS was first identified in SV40 T antigen as KKKRK [30,31]. This sequence is required for association with transport cargo protein importin α [26]. Furthermore, NLS is generally composed of a stretch of several positively charged amino acids, such as arginine (R) and lysine (K). Therefore, we searched for NLS-like amino acid sequence in the linker region of PNKP (Fig 2A). Consequently, we found a stretch of positively charged amino acids in the linker region of PNKP between amino acids 137–142 (KKRMRK), which is highly conserved in PNKP orthologs among a variety of mammalian species (Fig 2A). Additionally, this stretch showed high similarity with other known monopartite NLS motifs such as c-Myc, NAB2, H2B, SAMHD1, and SV40 (Fig 2B) [32–36]. To test whether the stretch of amino acids 137–142 is a functional NLS of PNKP, we constructed GFP-tagged PNKP 137–142 deletion mutant (Δ137–142) and transfected it into U2OS cells. Protein expression level of these constructs was confirmed by western blot analysis (Fig 2C).

As we expected, PNKP Δ137–142 mutant dramatically attenuated localization to the nucleus according to the live-cell imaging results (Fig 2D). To evaluate the nuclear localization ability of the mutant quantitatively, we measured GFI and analyzed the N/C ratio of the intensity (Fig 2E). As a result, PNKP Δ137–142 mutant (Mean of column: 0.7812) showed significant decrease of the ratio compared with FL PNKP (Mean of column: 6.210).

**Fig 3:**
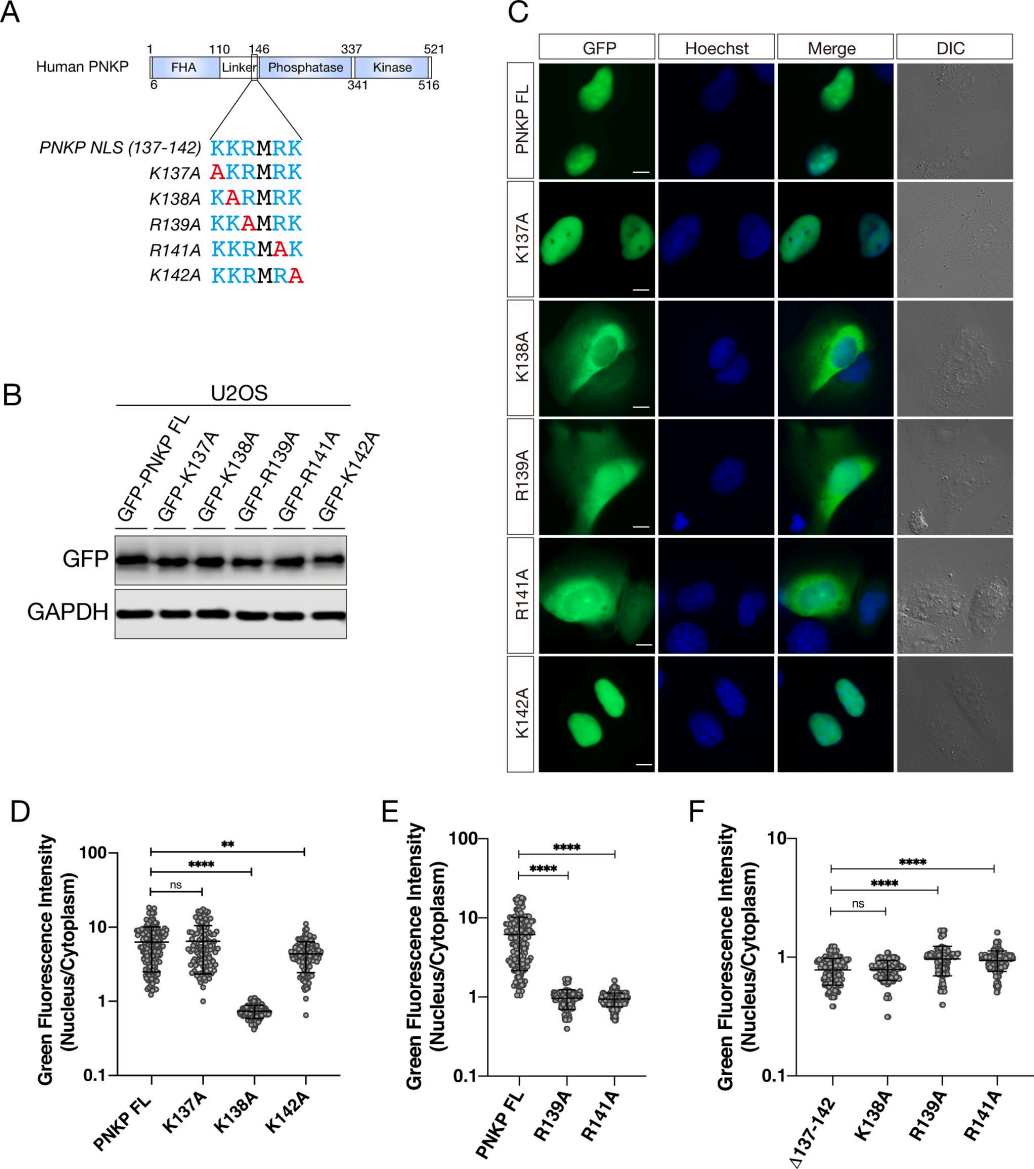
Lysine 138, arginine 139 and arginine 141 are the key residues for transportation into nucleus. (A) Diagram showing the amino acid substitutions between 137–142 in PNKP. Substituted amino acids were indicated by red. (B) Confirmation of protein expression of GFP-PNKP constructs in U2OS cells by western blot analysis. (C) Representative live-cell images of GFP-tagged NLS point mutated PNKP, expressing in U2OS cells, with DIC images. Scale bar indicates as 10μm. (D, E, F) Dot plot of the N/C ratio of GFI in FL and NLS point mutated PNKP. At least 150 cells were analyzed and plotted. ns: not significant (p > 0.05); **: 0.005 < p ≦ 0.01; ****: 0.0005 < p ≦ 0.001.

**Fig 4:**
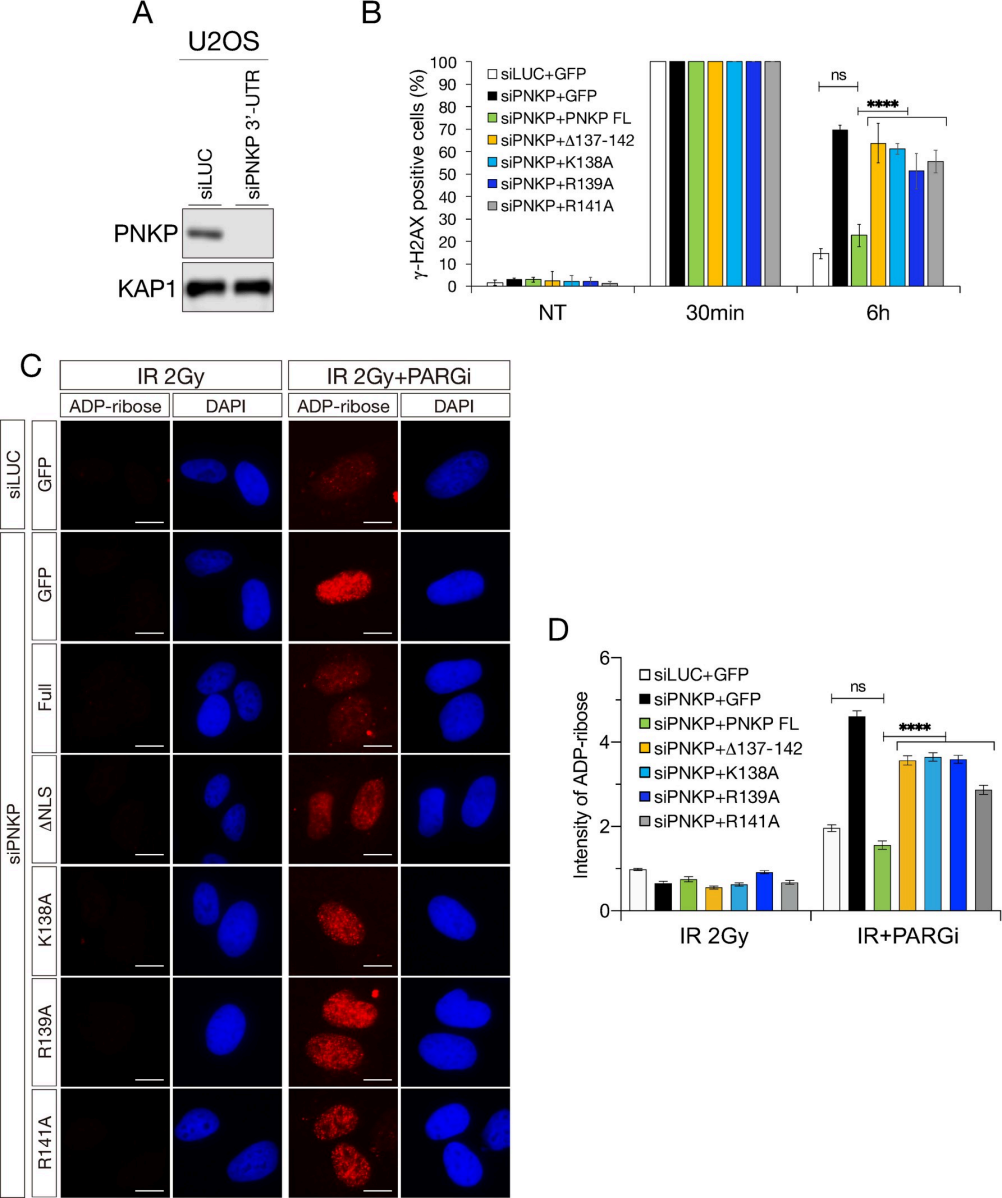
Lysine 138, arginine 139 and arginine 141 are important for efficient DNA repair. (A) PNKP depletion by 3’-UTR siRNA PNKP was confirmed by western blot analysis. KAP1 antibody was used for loading control. (B) DNA DSB repair efficiency was analyzed by immunofluorescence using γ-H2AX antibody after IR exposure in GFP-PNKP and 3’-UTR siRNA PNKP-transfected U2OS cells. Graph represented percentage of γ-H2AX positive cells with 10 ≧ γ-H2AX foci. At least 200 cells were counted and experiments were performed 3 times independently. (C) DNA SSB repair efficiency was analyzed by immunofluorescence using PAN-ADP-ribose binding reagents at 30 mins after IR exposure in GFP-PNKP and 3’-UTR siRNA PNKP-transfected U2OS cells treated with 10μM of poly (ADP-ribose) glycohydrolase inhibitor (PARGi) for 30 mins prior to IR exposure. (D) Graph represented the intensity of ADP ribose. At least 100 cells were counted and experiments were performed 3 times independently. Error bars indicate the standard error of the mean (SEM). ns: not significant (p > 0.05); **: 0.005 < p ≦ 0.01; ****: 0.0005 < p ≦ 0.001.

We further sought to identify the key residues for nuclear localization between amino acids 137–142 of PNKP. Since some reports suggested an importance of lysine and arginine in the NLS for efficient nuclear localization [37,38], we constructed three lysine- and two arginine-substituted PNKP mutants, *i*.*e*., K137A, K138A R139A, R141A and K142A, and transfected them into U2OS cells (Fig 3A). Protein expression level of these constructs was confirmed by western blot analysis (Fig 3B). In the same way as above, we also observed GFP-PNKP localization by live-cell imaging and analyzed the N/C ratio of the GFI (Fig 3C–3F). Interestingly, K138A mutant (Mean of column: 0.7883) showed similar level of attenuation of nuclear localization with Δ137–142 mutant (Fig 3D). Further, R139A (Mean of column: 0.9667) and R141A (Mean of column: 0.9431) mutants showed a greatly attenuated nuclear localization compared to PNKP FL, although they showed slightly but significantly higher nuclear localization than Δ137–142 mutant (Fig 3E and 3F). In contrast, K137A mutant (Mean of column: 6.434) did not show statistically significant difference compared with FL PNKP (Mean of column: 6.302) and K142A mutant (Mean of column: 4.384) showed mild attenuation in nuclear localization (Fig 3D). In addition, to analyze DNA repair efficiency, we performed immunofluorescence experiments using γ-H2AX antibody as the DNA double-strand breaks marker or a PAN-ADP-ribose binding regent as the DNA single-strand breaks marker [39–41] after IR exposure in U2OS cells (Fig 4A and 4B). PNKP depletion by 3’-UTR siRNA PNKP caused delay in DSB repair as compared to siLUC control cells (γ-H2AX positive cells were 69.8% and 14.6%, respectively). Whereas the expression of PNKP FL in PNKP depleted cells mostly restored efficient DSB repair (γ-H2AX positive cells were 22.6%), the expression of mutants in putative NLS did not (γ-H2AX positive cells were 63.7% in Δ137–142, 61.3% in K138A, 51.3% in R139A and 55.6% in R141A: Fig 3H). There was no significant difference between PNKP NLS mutants expressing cells in DNA DSB repair function. In the measurement of SSB repair efficiency, cells were pre-incubated with 10μM of PARG inhibitor (PARGi) 30 min prior to irradiation, incubation for short periods with PARGi was known to lead a significant increase in the ADP-ribose following IR exposure [39] (Fig 4C). PNKP-depleted cells showed defective SSB repair as compared with siLUC control cells (the mean of ADP-ribose intensity was 4.609 and 1.961, respectively). The re-expression of PNKP FL restored efficient SSB repair in PNKP-depleted cells (the mean of ADP-ribose intensity was 1.557), but the re-expression of putative NLS mutants partially did not (the mean of ADP-ribose intensity was 3.567 in Δ137–142, 3.647 in K138A, 3.591 in R139A and 2.869 in R141A) (Fig 4D). Taken together, amino acids 137–142 of PNKP are required for proper nuclear localization, and especially lysine 138, arginine 139 and arginine 141 are structurally important for association with transport factor and localization into the nucleus and efficient repair of both of DNA SSBs and DSBs.

### FHA domain of PNKP regulates subnuclear distribution

We have shown above that the nuclear localization of FHA deletion mutant (D1) of PNKP was slightly but significantly reduced as compared to FL PNKP (Fig 1), suggesting that the FHA domain as well as the NLS in the linker region is involved in the nuclear localization of PNKP. FHA domain, in general, mediates phosphorylation-dependent protein-protein interaction, and it has been known that FHA domain of PNKP mediates interaction with proteins such as XRCC1 and XRCC4 to accumulate to the DNA damage sites. It might be noted that XRCC1 has a bipartite NLS (244–248: KRKL; 268–276: VPKRPKLP) [42]. XRCC4 also has a monopartite NLS (270–275: RKRRQR) [43]. Thus, a possibility of PNKP nuclear translocation via association with XRCC1 or XRCC4 might be considered.

**Fig 5:**
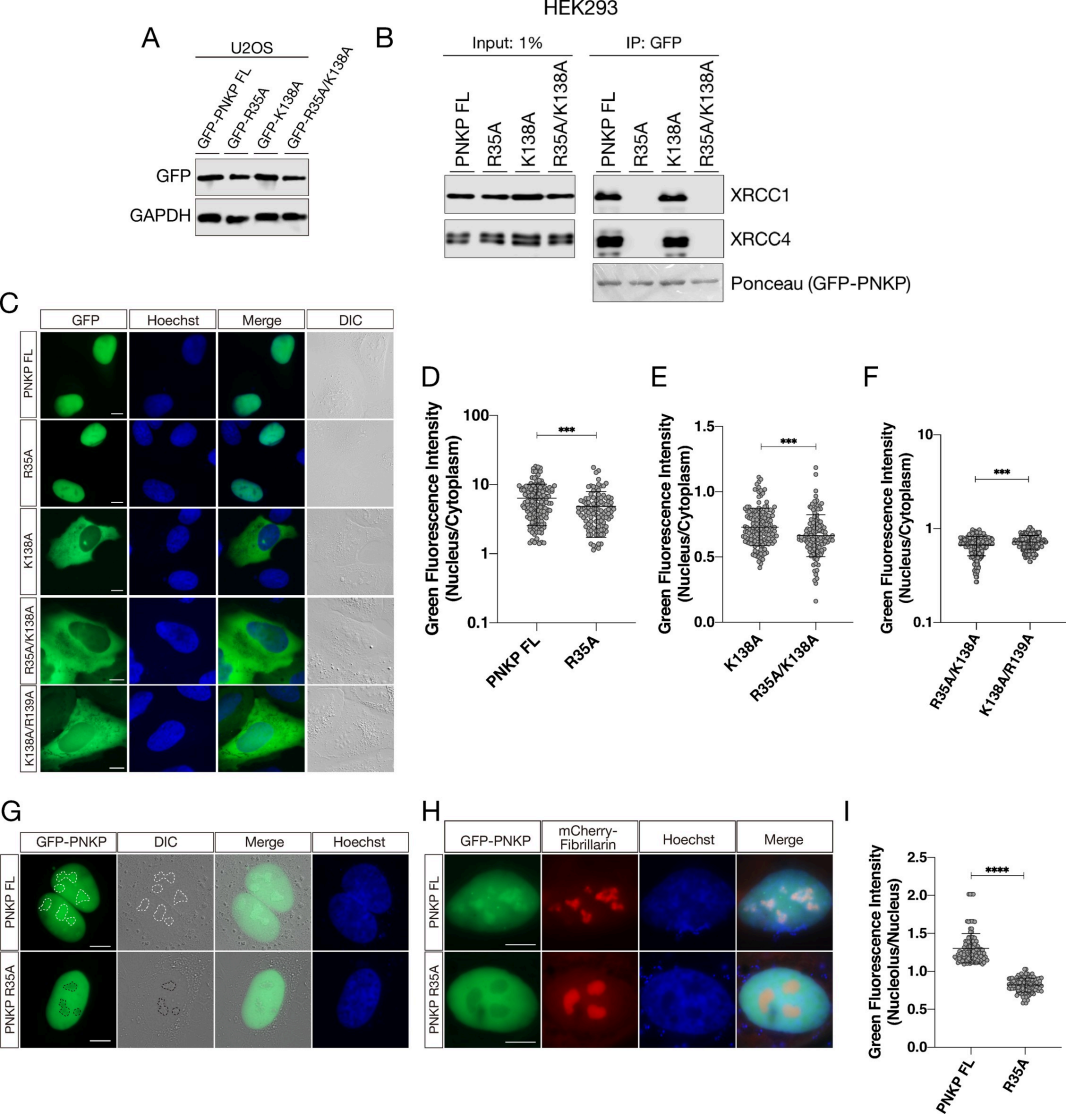
FHA domain of PNKP is involved in distribution to subnuclear compartments. (A) Confirmation of protein expression of GFP-PNKP constructs in U2OS cells by western blot analysis. (B) Interaction between FL PNKP and PNKP mutants (R35A, K138A and R35A/K138A) examined by immunoprecipitation. GFP-Trap magnetic agarose beads were used for immunoprecipitation from GFP-tagged PNKP expressing HEK293 cells. XRCC1 and XRCC4 were detected by western blotting and GFP-tagged PNKP was detected by Ponceau staining. (C) Representative live-cell images of GFP-tagged PNKP mutants, expressing in U2OS cells, with DIC images. Scale bar indicates as 10μm. (D, E, F) Dot plot of the N/C ratio of GFI. At least 150 cells were analyzed and plotted. (G) GFP-tagged FL PNKP and R35A PNKP localization in nucleus. Subnuclear regions with increased or decreased GFI are surrounded by white or black dashed circles, respectively. (H) Representative live-cell images of GFP-tagged FL PNKP, R35A PNKP and mCherry-tagged Fibrillarin localization in nucleolus. (I) Dot plot of the nucleous/nucleus ratio of GFI. At least 150 cells were analyzed and plotted. ns: not significant (p > 0.05); **: 0.005 < p ≦ 0.01; ***: 0.001 < p ≦ 0.005; ****: 0.0005 < p ≦ 0.001.

To further clarify the role of the FHA domain in the nuclear localization of PNKP, we constructed R35A mutant (Fig 5A). Arginine 35 is shown to be critically important for phosphorylation-dependent interaction with XRCC1 and XRCC4 [6,44]. Moreover, arginine residues corresponding to PNKP arginine 35 (R35) are highly conserved among the FHA domains of various proteins [20]. We also explored the functional relationship between NLS and FHA domain by analyzing K138A and R35A/K138A as well. Firstly, GFP-immunoprecipitation showed complete abrogation of binding ability of PNKP R35A and R35A/K138A with XRCC1 and XRCC4, whereas K138A mutant kept binding with them (Fig 5B). This result indicated that K138 is not required for interaction with XRCC1 and XRCC4 and that PNKP-XRCC1 and PNKP-XRCC4 complex can be formed in the cytoplasm. Moreover, this result also indicated that the interaction with XRCC1 or XRCC4 is not sufficient for proper nuclear localization of PNKP, despite that XRCC1 and XRCC4 have NLS, further substantiating the importance of PNKP’s own putative NLS. Live-cell imaging (Fig 5C–5F) showed that R35A (Mean of column: 4.840) and R35A/K138A (Mean of column: 0.664) mutants showed slightly but significantly attenuation nuclear localization compared with FL PNKP (Mean of column: 6.317), single K138A PNKP mutant (Mean of column: 0.7299), and double K138A/R139A mutant (Mean of column: 0.7205), respectively. Furthermore, we analyzed the distribution of FL PNKP and R35A PNKP mutant inside nucleus (Fig 5G). Interestingly, GFP-tagged FL PNKP showed strongly accumulation at several regions in nucleus. In contrast, GFP-tagged R35A PNKP was diminished at several regions in nucleus. From these results, R35 might be important for interaction with phosphorylated proteins to localize to subnuclear compartments such as nucleolus. To confirm this hypothesis, we constructed mCherry2-tagged nucleolus protein Fibrillarin expression vector. FL PNKP strongly accumulated with Fibrillarin in nucleus and R35A PNKP diminished from Fibrillarin accumulated region suggesting PNKP also function in nucleolus (Fig 5H). This observation suggested the possibility that protein interaction via FHA domain is required for the distribution of PNKP in the nucleus.

## Conclusion

PNKP possesses dual function as phosphorylation and dephosphorylation of DNA ends to facilitate DNA ligation. Since enzymatic activity of PNKP is important for DNA repair, especially for SSB repair and NHEJ for DSB repair, genetic mutation of *PNKP* gene result in inherited neuronal development and neurodegenerative disease. Previously, it is reported molecular interaction and regulation of PNKP with several DNA repair factors. Scaffold proteins XRCC1 and XRCC4 are important for the recruitment of PNKP to the DNA damage sites and phosphorylation of PNKP at serine 114 and serine 126 by ATM and DNA-PKcs is required for PNKP protein stability. However, it was unknown that how PNKP imported into nucleus from subcellular area. In this study, we identified putative NLS in the linker region of PNKP, and lysine 138, arginine 139 and arginine 141 therein which are essential for proper nuclear localization and efficient DNA repair. Moreover, we found that FHA domain of PNKP might regulate subnuclear distribution. Thus, this study revealed two distinct mechanisms regulating the PNKP distribution. These findings would contribute to deeper understanding of a variety of DNA repair pathway, *i*.*e*., BER, SSBR and DSBR.

## Supporting information

S1 Fig WB Full-scan data. (PDF)

S1 Table (XLSX)
