# Supplementary material for: Linker region is required for efficient nuclear localization of polynucleotide kinase phosphatase
Source: PLoS One. 2020 Sep 24;15(9):e0239404. doi: 10.1371/journal.pone.0239404 (PMC7514006; doi:10.1371/journal.pone.0239404)

Figure 1 WB Full-scan data

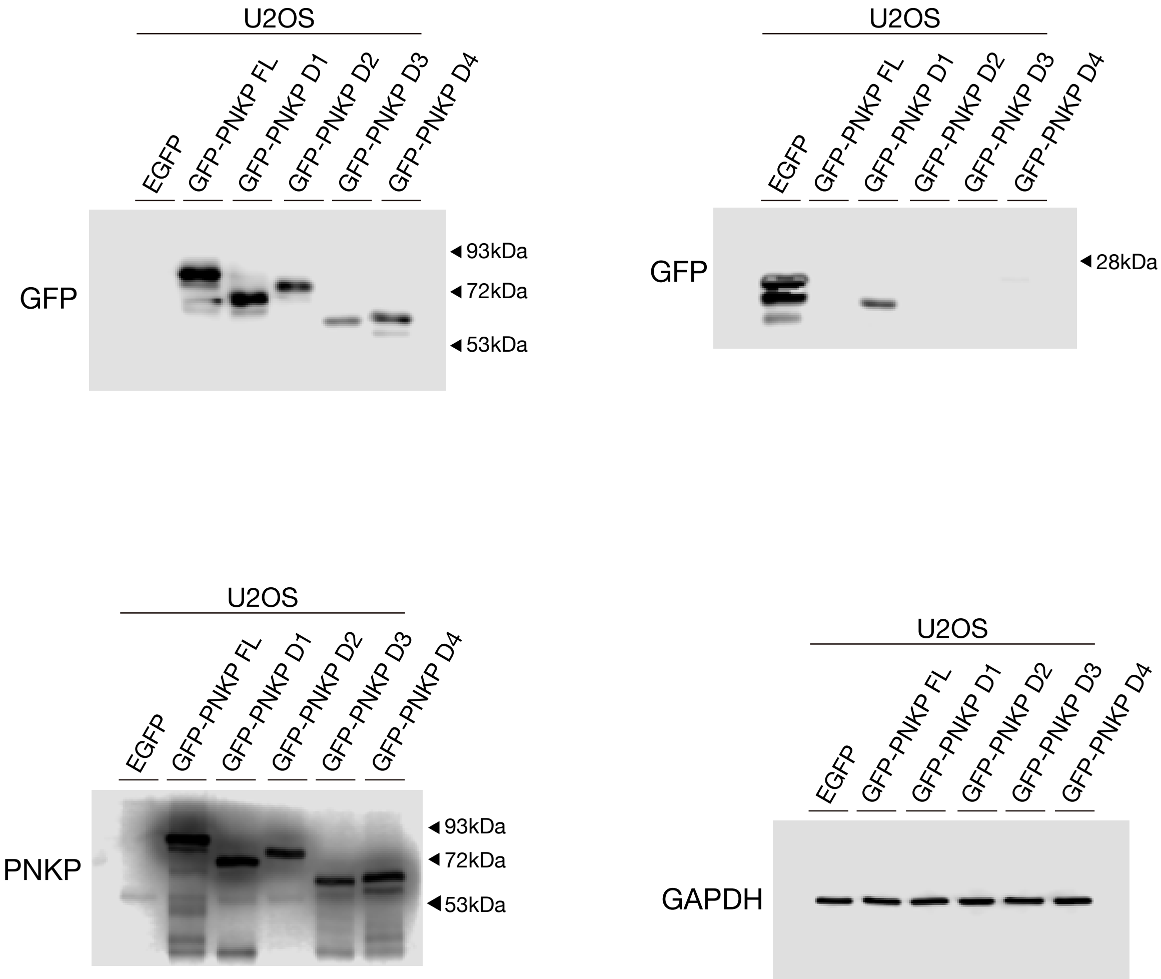

Figure 2 WB Full-scan data

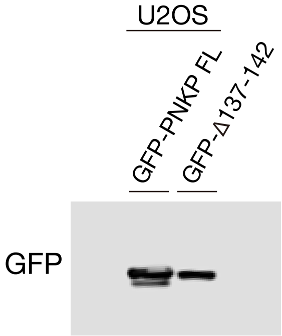

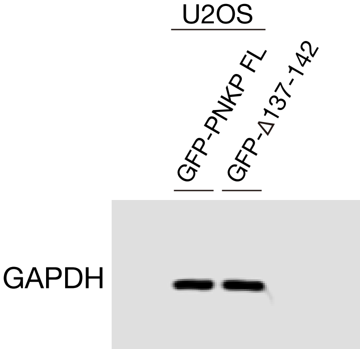

Figure 3 Full-scan data

B

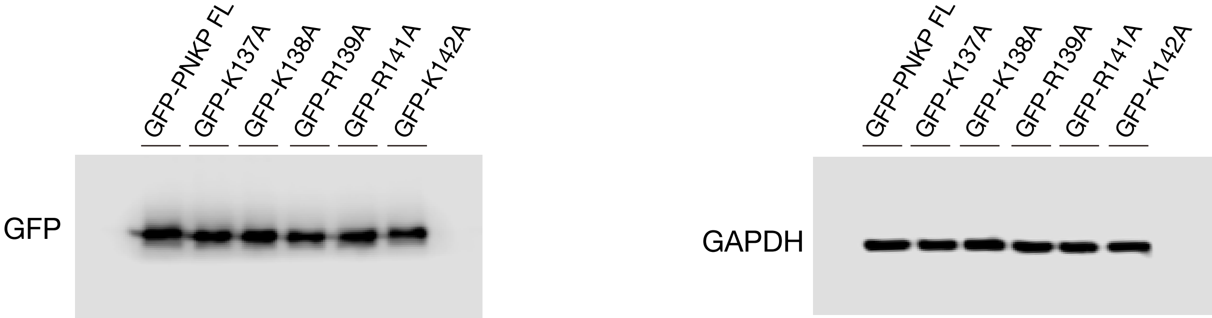

G

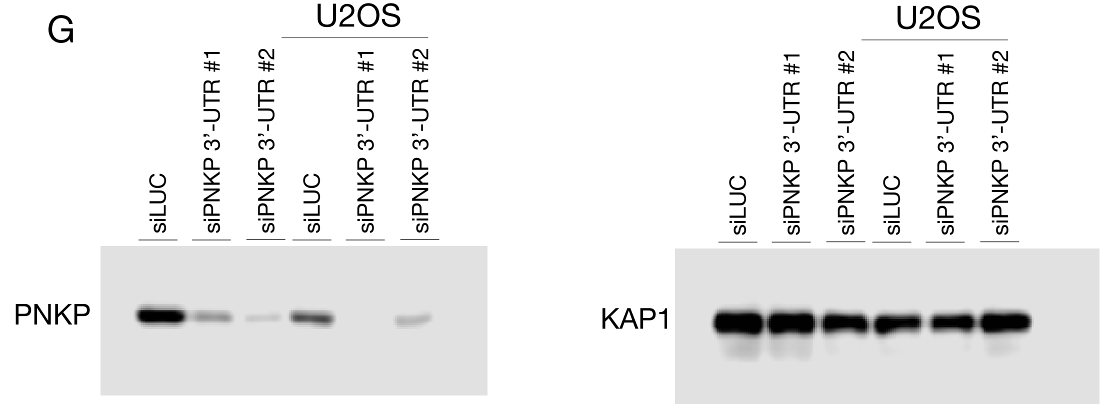

Figure 4 Full-scan data

A

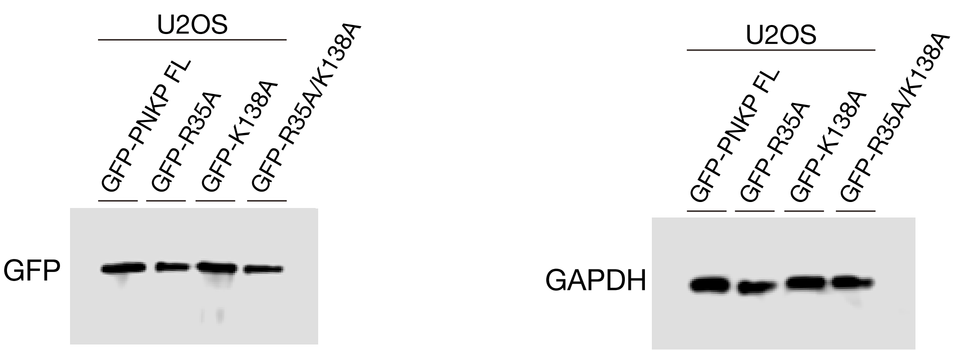

B

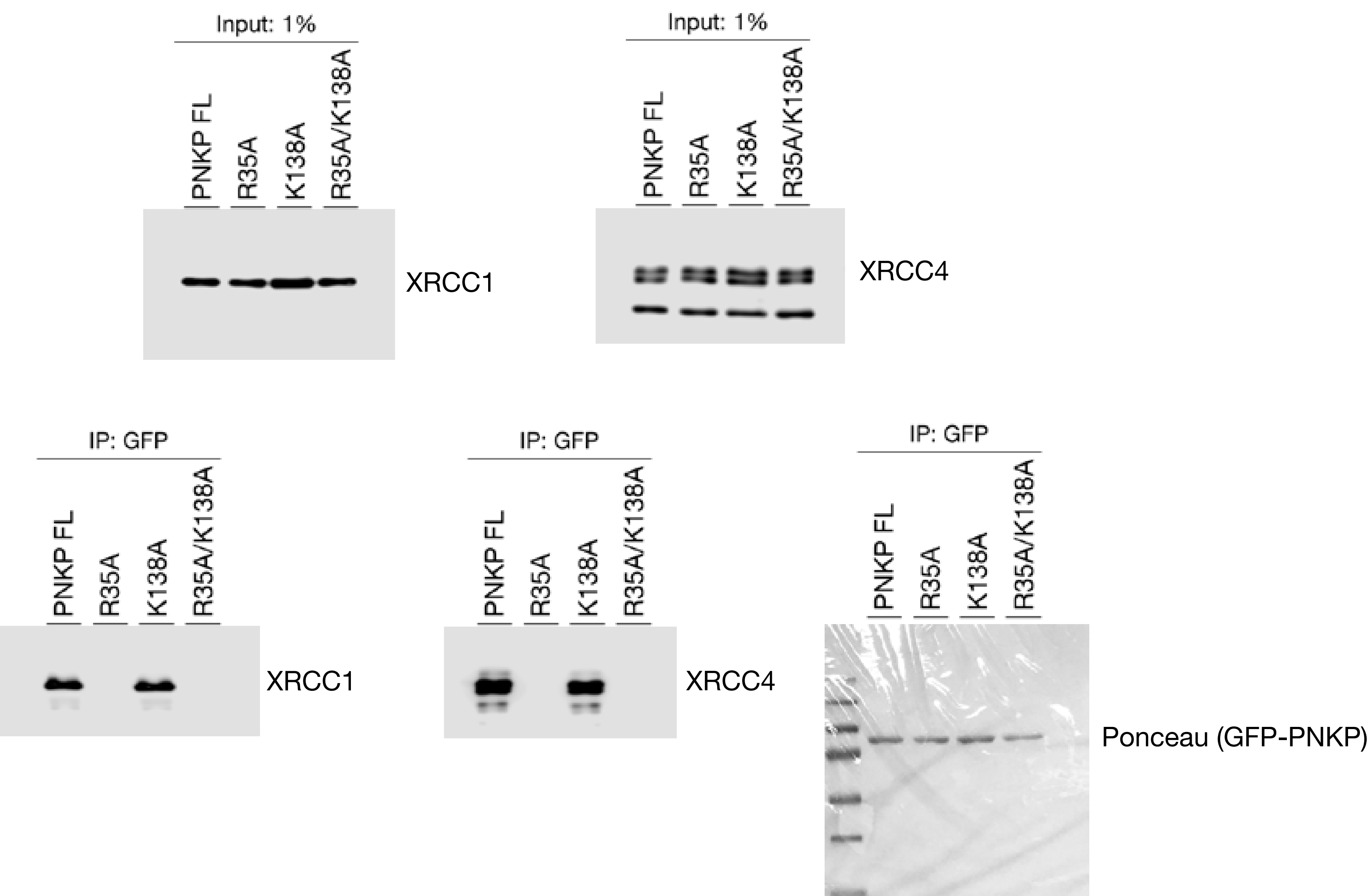

Supplement: S1 Fig — (PDF) [file pone.0239404.s001.pdf]
